# ^99m^Tc-labeled FAPI compounds for cancer and inflammation: from radiochemistry to the first clinical applications

**DOI:** 10.1186/s41181-024-00264-0

**Published:** 2024-05-02

**Authors:** Alessandra Boschi, Luca Urso, Licia Uccelli, Petra Martini, Luca Filippi

**Affiliations:** 1 Department of Chemical, Pharmaceutical and Agricultural Sciences, University of Ferrara, Via L. Borsari, 46, 44121 Ferrara, Italy; 2 Department of Translational Medicine, University of Ferrara, Via Fossato di Mortara, 70 c/o viale Eliporto, 44121 Ferrara, Italy; 3 Nuclear Medicine Unit, Ferrara Hospital, Via A. Moro, 8, 44124 Ferrara, Italy; 4 Department of Environmental and Prevention Sciences, University of Ferrara, Via L. Borsari, 46 44121 Ferrara, Italy; 5 Nuclear Medicine Unit, Department of Oncohaematology, Fondazione PTV Policlinico Tor Vergata University Hospital, Rome, Italy

**Keywords:** Molecular imaging, Fibroblast activation protein, Oncology, Inflammation, SPECT, PET

## Abstract

**Background:**

In recent years, fibroblast activating protein (FAP), a biomarker overexpressed by cancer-associated fibroblasts, has emerged as one of the most promising biomarkers in oncology. Similarly, FAP overexpression has been detected in various fibroblast-mediated inflammatory conditions such as liver cirrhosis and idiopathic pulmonary fibrosis. Along this trajectory, FAP-targeted positron emission tomography (PET), utilizing FAP inhibitors (FAPi) labeled with positron emitters, has gained traction as a powerful imaging approach in both cancer and inflammation. However, PET represents a high-cost technology, and its widespread adoption is still limited compared to the availability of gamma cameras. To address this issue, several efforts have been made to explore the potential of [^99m^Tc]Tc-FAPi tracers as molecular probes for imaging with gamma cameras and single photon emission computed tomography (SPECT).

**Main body:**

Several approaches have been investigated for labeling FAPi-based compounds with ^99m^Tc. Specifically, the mono-oxo, tricarbonyl, isonitrile, and HYNIC strategies have been applied to produce [^99m^Tc]Tc-FAPi tracers, which have been tested in vitro and in animal models. Overall, these labeling approaches have demonstrated high efficiency and strong binding. The resulting [^99m^Tc]Tc-FAPi tracers have shown high specificity for FAP-positive cells and xenografts in both in vitro and animal model studies, respectively. However, the majority of [^99m^Tc]Tc-FAPi tracers have exhibited variable levels of lipophilicity, leading to preferential excretion through the hepatobiliary route and undesirable binding to lipoproteins. Consequently, efforts have been made to synthesize more hydrophilic FAPi-based compounds to improve pharmacokinetic properties and achieve a more favorable biodistribution, particularly in the abdominal region. SPECT imaging with [^99m^Tc]Tc-FAPi has yielded promising results in patients with gastrointestinal tumors, demonstrating comparable or superior diagnostic performance compared to other imaging modalities. Similarly, encouraging outcomes have been observed in subjects with gliomas, lung cancer, breast cancer, and cervical cancer. Beyond oncological applications, [^99m^Tc]Tc-FAPi-based imaging has been successfully employed in myocardial and idiopathic pulmonary fibrosis.

**Conclusions:**

This overview focuses on the various radiochemical strategies for obtaining [^99m^Tc]Tc-FAPi tracers, highlighting the main challenges encountered and possible solutions when applying each distinct approach. Additionally, it covers the preclinical and initial clinical applications of [^99m^Tc]Tc-FAPi in cancer and inflammation.

**Supplementary Information:**

The online version contains supplementary material available at 10.1186/s41181-024-00264-0.

## Background

Fibroblasts are ubiquitous in the connective tissues of the human body. Although generally in quiescent status, these cells might be activated by the insurgence of damage in various circumstances, including wound healing, fibrosis, phlogosis, and cancer. In many cancer tissues, the unbalanced presence of inflammation and tissue damage determines activation of Cancer-Associated Fibroblasts (CAFs) that support tumor growth and evolve alongside tumoral cells (Dendl et al. 2021). Interestingly, CAFs overexpress fibroblast activating protein (FAP), which is therefore abundant in many cancer histotypes. Similarly, FAP is overexpressed by fibroblasts in inflammatory conditions and fibrosis, such as liver cirrhosis and idiopathic pulmonary fibrosis (Mori et al. 2023; Tatar et al. 2023).

The cutting-edge of nuclear medicine research has recently focused on radiolabeled FAP inhibitors (FAPi), that can target both oncological and inflammatory conditions, in a modern nuclear medicine theranostic approach. Indeed, FAP represents an optimal target for modern molecular imaging and therapeutic probes, due to its low/absent expression in normal tissues (Altmann et al. 2021). During the last few years, a multitude of radiolabeled FAPI compounds has been tested in preclinical and clinical trials. Most of these radiotracers has been labelled with gallium-68 (^68^Ga) for diagnostic purposes, with very encouraging results in multiple clinical scenarios, including digestive tract neoplasms, head and neck cancers and idiopathic pulmonary fibrosis (Dadgar et al. 2024; Kandathil and Subramaniam 2024; Mori et al., 2023; Wegen et al. 2022). However, [^68^ Ga]Ga-FAPi radiotracers are not free of limitations as an in-site germanium-68/gallium-68 generator is required for their production, due to the short half-life of ^68^Ga (68 min) that prevents the shipping model, which is feasible for fluorine-18 (^18^F). Therefore, only a few high-volume clinical-activity centers can afford to bear and amortize the costs related to the germanium-68/gallium-68 generator and to the necessary presence of an in-site radiopharmacy. Moreover, Positron Emission Tomography (PET) requires expensive hybrid tomographs to develop its full potential. Despite the widespread of these tomographs in the last decades, their availability still does not tie that of gamma cameras, particularly in developing countries (Lindner et al. 2020a).

The aforementioned limitations of [^68^ Ga]Ga-FAPi PET/CT induced a few researchers to investigate the possible role of [^99m^Tc]Tc-FAPi radiotracers that may be used for Single Photon Emission Computed Tomography (SPECT). Tchnetium-99m (^99m^Tc) possesses optimal nuclide characteristics with a half-life of 6 h and gamma radiation with an energy of 140 keV. Additionally, it exhibits a rich coordination chemistry, accommodating various oxidation states and donor atom sets. Its accessibility is convenient and cost-effective through a ^99^Mo/^99m^Tc generator. Many [^99m^Tc]Tc-labeled complexes can be synthesized through kit formulation, ensuring high radiochemical purity (RCP). The labeling process is straightforward, efficient, and reproducible, offering extensive potential applications in nuclear medicine diagnosis.

Aim of this review is to provide an updated state of art of approaches that have been investigated for labeling FAPi with ^99m^Tc and of [^99m^Tc]Tc-FAPi SPECT/CT, both on a preclinical and clinical perspective.

## Main text

### ^99m^Tc-labeling approaches

Technetium-99m (^99m^Tc), classified as a transition metal, faces a significant drawback compared to other radionuclides when it comes to bonding with biologically active molecules. Specifically, ^99m^Tc is unable to replace a carbon atom (e.g., carbon-11 (^11^C)) or a hydrogen atom (e.g., ^18^F and iodine-123 (^123^I)) within a targeting molecule. In order to incorporate a ^99m^Tc atom into the molecular structure of a biologically active molecule, it must go beyond the simple concept of labelling, understood as the substitution of an atom in the biomolecule with the radionuclide of interest, and refer to the principles governing coordination chemistry. Technetium utilizes coordination bonds to stable associate with a biomolecule. Different from a traditional covalent bond, which is established through the sharing of electron pairs between atoms, a coordination bond forms through the donation of an electron pair by a donor atom to a metal ion. This pair of electrons is accepted by the metal, acting as a Lewis acid, thus giving rise to a new chemical species known as a coordination complex. The biologically active molecule must therefore be associated with the chemical species (ligand) that contains the atom or atoms able to coordinate technetium. The nature of the donor atoms specifically determines the coordination number, defined as the number of donor atoms surrounding the metal, and the geometry of the resulting complex. The coordination chemistry of technetium is notably rich, characterized by a wide variety of geometries with the metal in several oxidation states ranging from + 1 to + 5 (Additional file 1: Fig. S1) (Bolzati and Dolmella 2020; Jones and Davison 1982). This is a particular advantage offered by technetium since the coordination number and geometry are fundamental parameters to consider in the design of metal complexes involving biomolecules. These parameters impart specific characteristics of lipophilicity and charge to the resulting chemical species, significantly influencing the in vivo localization and kinetics of the radiopharmaceutical. The choice of the coordinating system allows for the design of complexes with targeted pharmacokinetic properties.

^99m^Tc, is generally produced through a ^99^Mo/^99m^Tc generator system in the form of [^99m^Tc][TcO_4_]^−^ in a physiological solution. Therefore, to associate the radionuclide to a bioactive molecule, the Tc(VII) metal must undergo reduction to an appropriate oxidation state in the presence of suitable ligands as schematized in Additional file 1: Fig. S2. A profound understanding of coordination chemistry enabled the development of practical methods to reduce and incorporate stable technetium-99m into a bioactive molecule without compromising its bioactivity.

**Fig. 1:**
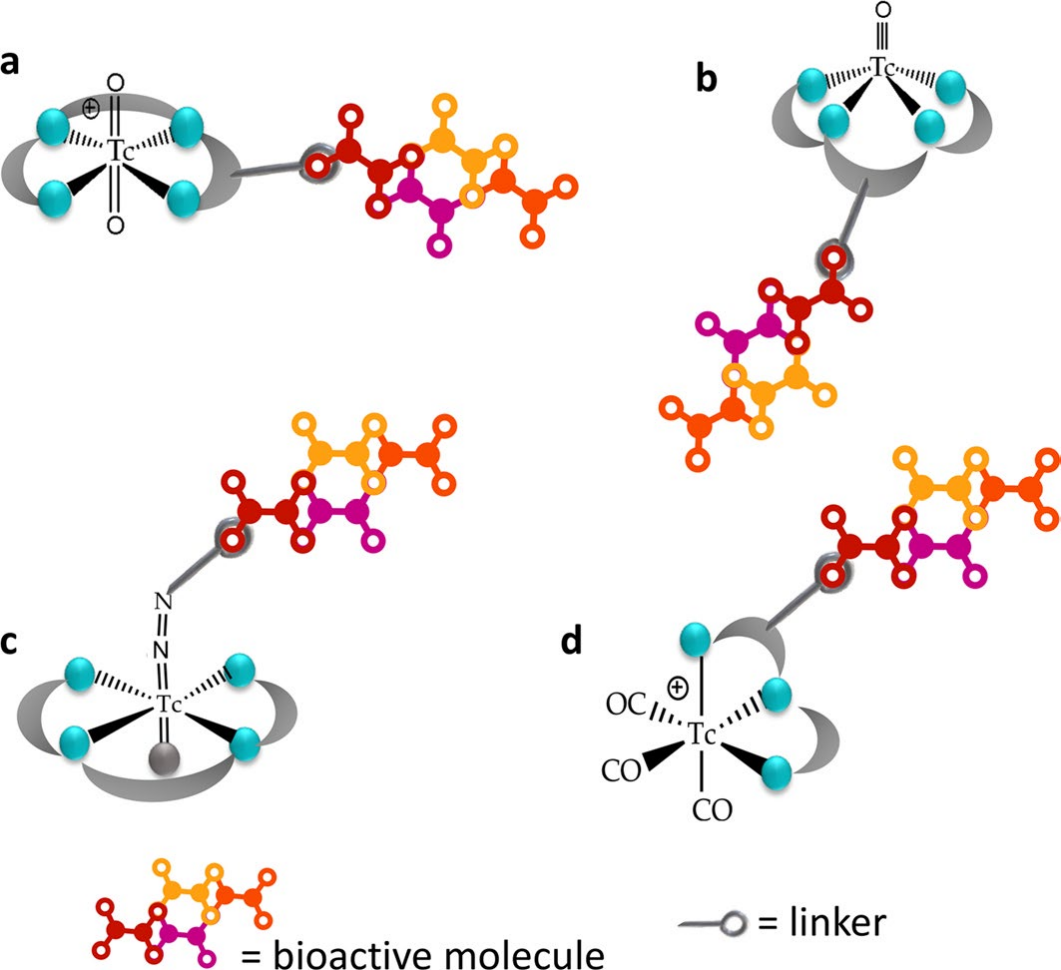
Some examples of bioactive molecule associated to different technetium-99m cores: **a**, di-oxo TcO_2_^+^ core; **b**, mono-oxo TcO^+^ core; **c**, Tc-HYNIC core; **d**, Tc(CO)_3_^+^ core

A possible strategy involves the use of an inorganic functional group of technetium. Several inorganic technetium functional groups, referred to as "cores" or "metal fragments," have been identified thus far. These functional groups can be synthesized in a physiological solution, featuring technetium in a reduced oxidation state and possessing labile coordination positions. These characteristics can be conveniently leveraged to incorporate the chosen bioactive molecule (Fig. 1).

In the following paragraphs, the potentially usable synthesis strategies for labeling bioactive molecules, such as FAPI, with ^99m^Tc are described. The [^99m^Tc]Tc-FAPI complexes developed using some of these strategies will be discussed in Sect. "^99m^Tc-FAPI labeled compounds".

### The mono-oxo [^99m^Tc][TcO]^3+^ and di-oxo [^99m^Tc][TcO_2_]^+^ cores

The [^99m^Tc][TcO]^3+^ core stands out as the most extensively researched metal fragment. Complexes based on this core are generally stabilized by molecules that include coordinating atoms such as N, S and to a lesser extent O. They typically exhibit a pentacoordinated structure, adopting a square pyramidal geometry with the π-bonding oxo-group positioned apically. The strong trans influence of the oxo-group makes six-coordinated technetium-oxo compounds relatively uncommon. The stability of the oxo-core is achieved through the involvement of σ- and π-donating atoms derived from amino, amido, and thiolate ligands. Additionally, tetradentate ligands of the N4-xSx class have been extensively explored in this context and include diamino-dithiols (DADT), diamido-dithiols (DADS), monoamino-monoamido-dithiols (MAMA), and the tripeptide mercaptoacetyltriglycine (MAG3). DADT and MAMA form neutral mono-oxo complexes with the metal, exhibiting pronounced lipophilicity. Chelators of the DADS and MAG3 type, on the other hand, give rise to anionic complexes that exhibit a pronounced renal clearance (Van Domselaar et al. 2000; Lei et al. 1996; Zhu et al. 2001).

Mixed amino-thiol-based chelators, particularly N_2_S_2_ and N_3_S, have been widely employed for labeling a selected bioactive molecule with ^99m^Tc (Hnatowich et al. 1998; Hom et al. 1996; Oya et al. 1998; Skaddan et al. 2000). Representative examples from this class of compounds include ^99m^Tc-TRODAT-1, a probe for diagnosing Parkinson’s disease, and ^99m^Tc-Depreotide (P829), a Tc(V)-oxo somatostatin analog complex (Kung et al. 1996; Menda and Kahn 2002).

The di-oxo core [O=Tc=O]^+^ is stabilized by ligands containing N (e.g., cyclic or linear tetraamines, N_4_) and to a lesser extent P (e.g., bis-phosphines, P_2_). With these ligands, it forms hexacoordinated, charged complexes with an octahedral geometry (Fig. 1). Complexes with tetradentate ligands of the N_4_, P_2_N_2_, or P_2_S_2_ type have been used for the labeling of biomolecules, peptides, and antibody (Bolzati et al. 2012). It should be emphasized that the inclusion of the [O=Tc(V)=O] + core in a bioactive molecule may contribute to enhancing the hydrophilicity of the originally lipophilic moiety, promoting excretion via the kidneys and the urinary system (Maina et al. 2002).

The limitations of these cores include the instability of the metal-chelate complex, with possible in vitro and in vivo oxidation of the metal, the purity of the radiolabeled product, the formation of diastereoisomers, and the lipophilic nature of both the ligand and the final complex, which negatively affects the pharmacokinetics of ^99m^Tc-radiopharmaceutical. Furthermore, another limiting factor is the low specific activity of the compounds, necessitating high concentrations of the ligand (10^–4^—10^–5^ M) to prepare complexes with high radiochemical purity. This can potentially lead to the saturation of the molecular target or induce pharmacological effects upon administration (Todde and Boschi 2020).

### The[ ^99m^Tc]Tc-HYNIC core

The HYNIC core is formed by a monodentate diazenido unit ([^99m^Tc]Tc=N=N-R^2+^) (Additional file 1: Fig. S1), that can easily be prepared from the [^99m^Tc][TcO]^3+^ core by a simple condensation reaction with the 6-hydrazinopyridine-3-carboxylic acid (HYNIC) (Banerjee et al. 2005). HYNIC forms a strong bond with the metal in the V oxidation state, creating a robust core occupying the ligand with one or two coordination sites. The coordination sphere of the metal is saturated by using co-ligands of different natures, such as EDDA (ethylenediamine-N,N'-diacetic acid), tricine, glucoheptonate, mannitol, thiolates, and phosphines can form various binary and ternary systems (Liu 2005).

The HYNIC ligand is particularly advantageous for radiolabeling biomolecules with ^99m^Tc because it combines simultaneously sites that are useful for stable coordination to the metal and a carboxylic function that can be skillfully exploited for condensation with the biomolecule of interest (Fig. 1). However, although the metal-organohydrazino core can be easily prepared by a two-vial kit formulation, the intricate details of the chemistry are more complex and contingent upon reaction conditions and the presence of co-ligands. These factors can influence the stability, hydrophilicity, and pharmacokinetics of the resulting ^99m^Tc-radiopharmaceuticals. The dependency of coordination chemistry on the reaction conditions has resulted in certain challenges in the development of ^99m^Tc-HYNIC radiopharmaceuticals. Furthermore, the increasing regulatory requirements for providing fully-characterized products do not seem to have facilitated the inclusion of HYNIC in clinical practice. Nevertheless, a broad range of bioactive molecules have been labeled using the HYNIC strategy, with somatostatin derivatives for neuroendocrine tumors (NETs) imaging being among the most extensively investigated (Liepe and Becker 2018; Trogrlic and Težak 2017).

The [^99m^Tc]Tc-HYNIC technology was employed in the development of a prostate-specific membrane antigen (PSMA) inhibitor for ^99m^Tc-based SPECT. In vitro and in vivo studies of [^99m^Tc]Tc-EDDA-HYNIC-iPSMA, rapidly prepared from a kit formulation with a radiochemical yield exceeding 95%, demonstrating that this radiopharmaceutical could detect tumors and metastases of prostate cancer (PCa) similar to [^68^Ga]Ga-PSMA-617 (Ferro-Flores et al. 2017). The efficacy of [^99m^Tc]Tc-HYNIC-PSMA SPECT/CT in detecting primary lesions and metastases in newly diagnosed prostate cancer has recently been demonstrated (Wang et al. 2023).

### The [^99m^Tc][Tc(CO)_3_]^+^ core

The [^99m^Tc][Tc(CO)_3_]^+^ core (Additional file 1: Fig. S1) is a chemically robust organometallic structure consisting of technetium I coordinated to three carbonyl groups. The precursor [^99m^Tc][Tc(CO)_3_(H_2_O)_3_]^+^ can be easily prepared from the generator-eluted [^99m^Tc][TcO_4_]^−^ in a single step procedure under reducing conditions by potassium boranocarbonate K_2_[BH_3_CO_2_], that also acts as a source of carbonyl ligands as developed by Alberto et al. (Alberto et al. 1999). The three water molecules of the hexacoordinated, monocationic [^99m^Tc][Tc(CO)_3_(H_2_O)_3_]^+^ synthon can be easily replaced by bi- or tri-dentate ligands (L), predominantly possessing nitrogen as donor atoms. This results in a series of heteroleptic complexes, such as [^99m^Tc][Tc(CO)_3_(L)Cl] and [^99m^Tc][Tc(CO)_3_(L)]. Essentially all types of donor atoms have been used and numerous examples of bidentate and tridentate chelators have been reported, including derivatives that can be linked to targeting molecules (Fig. 1) (Mindt et al. 2006). In particular, it has been observed that the amino acid histidine exhibits high selectivity for the [^99m^Tc][Tc(CO)_3_]^+^ fragment and readily replaces water molecules even at very low concentrations (10^–5^–10^–6^ M). Histidine (his) derivatives can be easily linked to biomolecules by introducing an acetic acid group at N in histidine, resulting in an N carboxylate histidine that is ready to be condensed to a selected biomolecule. The use of histidine has the additional advantage of not being sensitive to reduction, and the resulting [^99m^Tc][Tc(CO)_3_his]^+^ can be prepared in a single reaction step from the generator-eluted [^99m^Tc][TcO_4_]^−^. Unfortunately, the partial replacement of aqua ligands promotes the substitution with more reactive blood proteins within the body, consequently altering the pharmacokinetic profiles and leading to elevated retention of the radiolabeled substance in the liver and kidneys (Schibli and Schubiger 2002). The use of polar tridentate ligands, that contain bis((1-(carboxymethyl)-1H-imidazol2-yl)methyl)amino donor group for chelation of the [^99m^Tc][Tc(CO)_3_]^+^, demonstrated reduced lipophilicity when conjugated to small molecules and peptides, such as Octreotide™, dramatically enhanced renal clearance and diminished hepatobiliary uptake (Maresca et al. 2010).

This strategy has been recently used for the development of a promising compound, [^99m^Tc]Tc-MIP-1404 (^99m^Tc-Trofolastat) based on an imidazole modification of the PSMA inhibitor (Hillier et al. 2013).

### The [^99m^Tc][TcN]^2+^ core

The nitrido [Tc(V)≡N]^2+^ core, provides an alternative building block to associate a biomolecule to technetium, ensuring superior stability to the resulting radiopharmaceutical under in vivo oxidizing-reducing conditions. Since 1990, Duatti and colleagues have successfully generated ^99m^Tc-species containing the terminal Tc≡N multiple bonds in sterile and pyrogen-free conditions, albeit at tracer levels (Duatti et al. 1990). In general, Tc(N)-complexes adopt a five-coordinate geometry, where one coordination site is taken up by the nitrido nitrogen atom, and the remaining positions are available for binding four donor atoms. Symmetrical complexes of the type [Tc(N)(L)_2_] (where L is a dithiocarbamate or phosphinothiol ligand) and mixed nitrido compounds of the type [Tc(N)(X)(Y)] (where X and Y are two different polydentate ligands) have been reported (Bolzati et al. 2010; Bolzati et al. 2000; Bolzati et al. 2000). The mixed nitrido compounds' characteristics provide an intriguing foundation for labeling bioactive molecules. In particular, the production of [Tc(N)(X)(diphosphinoamine)]^0/+^ (diphosphinoamine = PNP) complexes relies on the chemical characteristics of the electrophilic nature of the [Tc(N)(PNP)]^2+^ building block, that selectively reacts with ligands (X) containing soft π-donors coordinating atoms (S, O, N) to yield the ultimate mixed compound. It was determined that naturally occurring bidentate ligands, such as cysteine, exhibited excellent coordinating properties toward the [Tc(N)(PNP)]^2+^, offering a straightforward method for incorporating biomolecules into a ^99m^Tc(N) asymmetrical complex (Bolzati et al. 2003; Boschi et al. 2003).

**Fig. 2:**
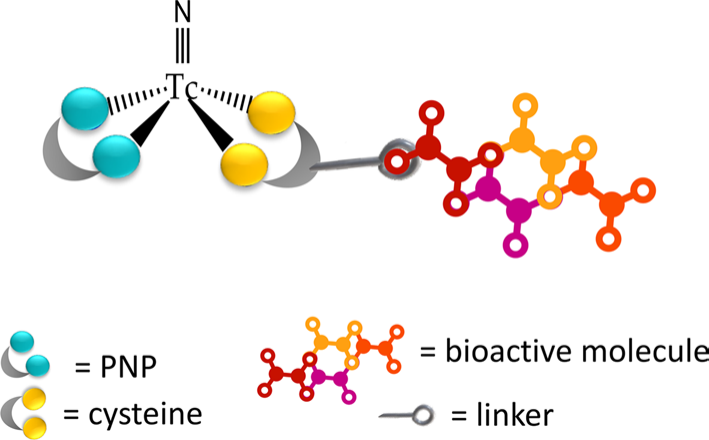
Schematic representation of the labeling of a bioactive molecule using the [Tc(N)(PNP)]^2+^approach to give the [^99m^Tc][Tc(N)PNP-Cys-biomolecule]^0/+^ complex

Cysteine (Cys) binds with high specific activity (70 GBq/μmol) to the [Tc(N)(PNP)]^2+^ moiety either through the [NH_2_,S^−^] pair or, alternatively, the [O^−^, S^−^] pair of donor atoms, resulting in the formation of the corresponding mixed compound. In both cases, cysteine can be chemically linked to a biomolecule either through the C-terminal or N-terminal residue group to afford the mixed [^99m^Tc][Tc(N)PNP-Cys-biomolecule]^0/+^ complex as schematized in Fig. 2. Generally, the [^99m^Tc][Tc(N)PNP-Cys-biomolecule]^0/+^ complex can be easily prepared in high radiochemical purity and with a good specific activity through a two steps reaction suitable for a two-vial kit formulation. In the first step the generator eluted [^99m^Tc][TcO_4_]^−^ is added to a vial containing succinic dihydrazide (SDH) as nitrido-donor ligand and SnCl_2_ as reducing agent to produce at room temperature the [^99m^Tc][TcN]^2+^ core. Following the reaction, PNP and the Cys-biomolecule ligands are added to give the final [^99m^Tc][Tc(N)PNP-Cys-biomolecule]^0/+^ compound. Additional advantages of this technology include, the high in vivo stability of the resulting complexes and the well-defined chemical nature of the complexes that can be determined using traditional chemical characterization techniques. This synthetic approach has been applied for the preparation of receptor-specific radiopharmaceuticals for imaging benzodiazepine receptors and receptor-specific tracers for 5HT1A receptors (Bolzati et al. 2003; Boschi et al. 2003).

The [^99m^Tc][TcN]^2+^ core has been also used for labeling bioactive molecules using the so-called “3 + 1” strategy. It is based on the combination of tridentate and monodentate ligands bound to the [Tc≡N]^2+^ group (Boschi et al. 2013, 2012). In this specific synthetic strategy, the tridentate ligand, conveniently created by pairing amino acids or pseudo-amino acids, such as cysteine–cysteine, cysteine–isocysteine, or cysteine–mercaptoacetic acid, binds the metallic core through a set of three π-donor atoms. The coordination sphere of the pentacoordinate geometry is saturated by the coordination of the monodentate ligand through a single π − acceptor atom, such as a phosphorus atom. This outcome could offer an almost 'natural' and straightforward approach to labeling peptides (or the selected biomolecule (Smilkov et al. 2014)) with ^99m^Tc, as it only necessitates extending the original peptidic sequence with a pair of chelating amino acids.

### The ^99m^Tc “4 + 1” approach

**Fig. 3:**
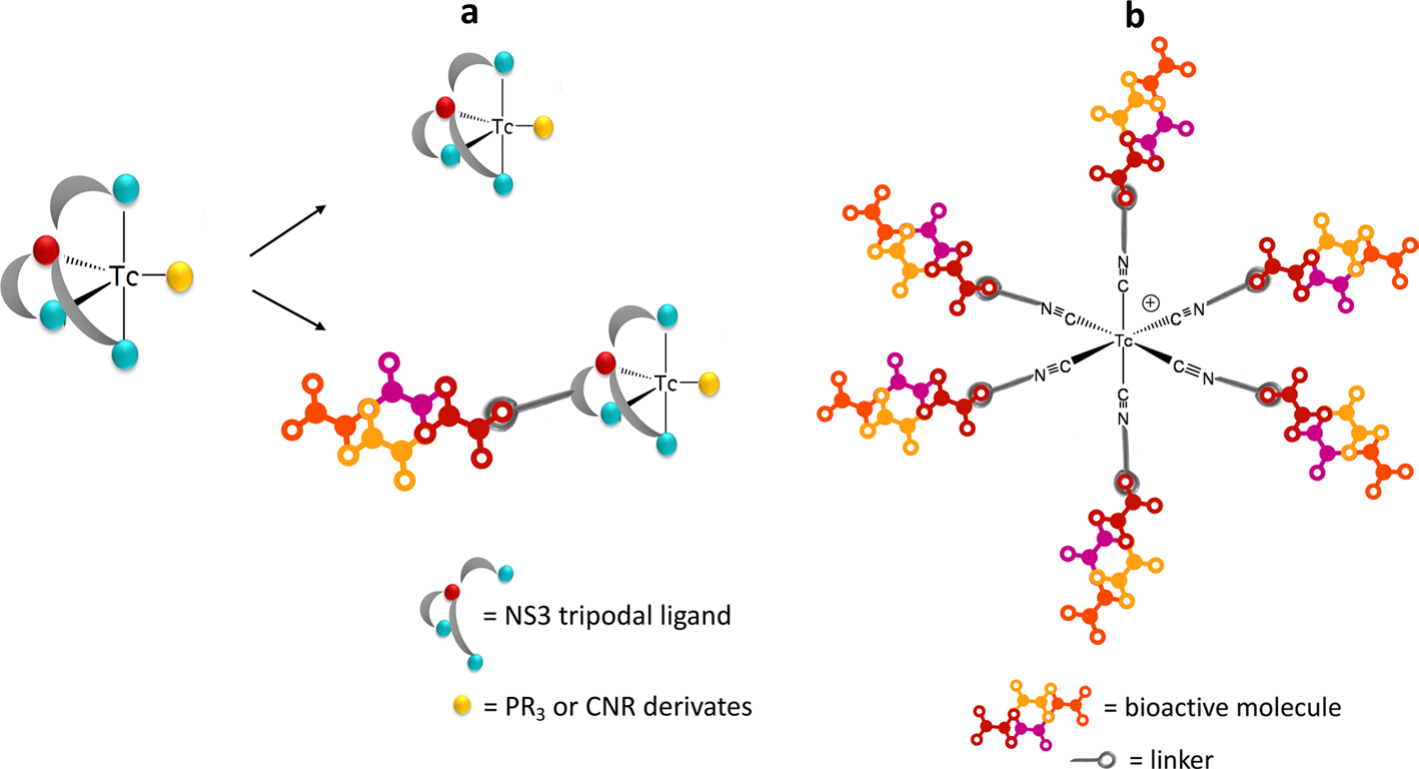
Schematic representation of the bioactive molecules labeling using: **a**, the ^99m^Tc “4 + 1” approach; **b**, bifunctional monodentate isonitrile ligands to give the [^99m^Tc][Tc(I)(CN-biomolecule)_6_]^+^ complex

The '4 + 1' approach involves the coordination of a tri-negative tripodal tetradentate 2,2',2"-nitrilotris-ethanethiol- (NS_3_) ligand and either a monodentate π-acceptor ligand, such as tertiary phosphine (PR_3_), or isocyanide derivatives (CNR), to a Tc(III) center (Seifert et al. 2004). The '4 + 1' complexes offer two distinct sites for the conjugation of a biomolecule. It can be linked either to the NS_3_ tripodal ligand (Fig. 3a) or to the isocyanide or phosphino co-ligand (Fig. 3a) by introducing lateral carboxylic groups. Labeling with ^99m^Tc was conducted through a two-step procedure, which helped avoid the production of reduced hydrolyzed metallic species. The initial step involved the preliminary formation of the intermediate complex [^99m^Tc]Tc(III)-EDTA by adding a fresh pertechnetate solution to a kit containing EDTA, mannitol, and SnCl_2_. In the second step, the [^99m^Tc]Tc(III)-EDTA species was reacted with the appropriate tripodal ligand as an oxalate salt and the monodentate one to produce, in 30 min at 75 °C, the ^99m^Tc ‘4 + 1’ complex in high radiochemical yield. Examples of small biomolecules labeled using the ^99m^Tc '4 + 1' approach include 2-methoxyphenylpiperazine, neurotensin and bombesin derivatives (Drews et al. 2002; Kunstler et al. 2007).

### The ^99m^Tc-isonitril strategy

Isonitrile ligands C≡N-R (where R is a general functional group) can coordinate and stabilize technetium in a low oxidation state to form highly stable monocationic six-coordinated homoleptic compounds of Tc(I). Well-known is the radiopharmaceutical for myocardial perfusion imaging [^99m^Tc]Tc-Sestamibi, which is based on the coordination of six methoxyisobutylisonitrile to Tc(I) (Boschi et al. 2022).

Very recently, isonitrile ligands have been proposed as bifunctional monodentate ligands for the labelling of bioactive molecules with technetium. Complexes of the type [^99m^Tc][Tc(I)(CN-R)_6_]^+^ (R is a functional group associated with the selected biomolecule) have been developed. Because the ^99m^Tc-labeled isocyanate-based complex featured six isonitrile ligands, each containing a targeting group, its ability to target the specific site could be improved (Fig. 3b). Only a few non-exhaustive examples are the preparation and the evaluation of [^99m^Tc]Tc-CNGU as a PSMA-targeted radiotracer for the imaging of prostate cancer (Xiao et al. 2020) and the preparation and evaluation of novel folate isonitrile ^99m^Tc-complexes as potential tumor imaging agents to target folate receptors (Feng et al. 2021). The preparation of [^99m^Tc][Tc(I)(CN-R)_6_]^+^ compounds is very simple occurring in a single reaction step without the need for further purifications (Feng et al. 2021).

## ^99m^Tc-FAPI labeled compounds

To the best of our knowledge, four out of the different synthetic approaches illustrated earlier have been applied for the FAPI labeling with ^99m^Tc: the mono-oxo, the tricarbonyl, the isonitrile and the HYNIC strategy.

In 2020 Roy et al. (2020) were the first to report a FAP-targeted ^99m^Tc imaging agent. [^99m^Tc]Tc-FL-L3 was prepared by conjugating the ligand FAPI-L3 to the tripeptide mercaptoacetyltriglycine (MAG3) chelator to label the [^99m^Tc][TcO]^3+^ core, while 8-amino-octanoic acid was used as the linking agent connecting the metal chelator and the FAP inhibitor (Additional file 1: Fig. S3). [^99m^Tc]Tc-FAPI-L3 exhibited strong binding and specificity for FAP in the FAP-transfected human embryonic kidney HEK 293 cell line. When tested in vivo on mice bearing MDA-MB231 breast tumors, [^99m^Tc]Tc-FAPI-L3 accurately delineated the experimental tumor with minimal uptake in non-target tissues. [^99m^Tc]Tc-FAPI-L3 was observed to accumulate in MDA-MB231 solid tumors (2.23% ID/g) and a co-injection of excess of FAPI was seen to block this tumor uptake (0.13 ± 0.06%ID/g), suggesting that tumor retention was FAP-mediated.

**Fig. 4:**
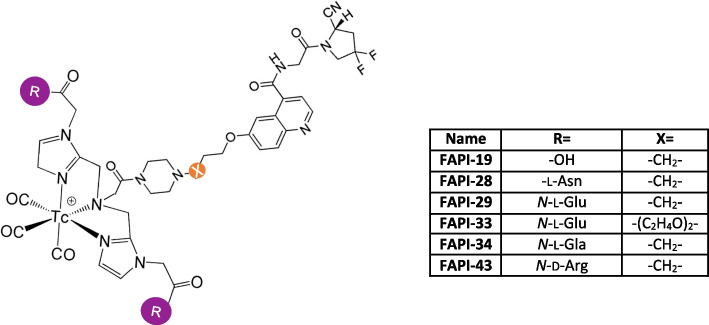
Chemical structure of ^99m^Tc-tricarbonyl-FAPI complexes

In 2020 Lindner and co-worker developed ^99m^Tc-tricarbonyl-FAPI complexes of the type shown in Fig. 4 (Lindner et al. 2020b). Despite employing polar tridentate ligands with a bis((1-(carboxymethyl)-1H-imidazol2-yl)methyl)amino donor group for chelation of [^99m^Tc][Tc(CO)_3_]^+^, the significant lipophilicity of the tricarbonyl complex, as observed in PSMA ligands or somatostatin receptor–targeted compounds (Lu et al. 2013; Maresca et al. 2010), leads to hepatobiliary elimination of FAPI-19, resulting in a limited tumor accumulation. This phenomenon could arise from non-specific binding of blood components, such as lipoproteins, which may overshadow the binding to FAP, hindering its conversion into the tumor tissue. This is accompanied by a rapid deposition rate in the liver without engaging in enterohepatic circulation. To enhance the pharmacokinetic properties, hydrophilic amino acids were incorporated into the chelator moiety of the compound. The resulting ^99m^Tc-labeled FAPI tracers exhibited outstanding binding properties (45% binding; > 95% internalization), high affinity (half-maximal inhibitory concentration, 6.4–12.7 nM), and notable tumor uptake (about 5.4% injected dose per gram of tissue) in biodistribution studies. The leading candidate, [^99m^Tc]Tc-FAPI-34, was employed for diagnostic scintigraphy and SPECT imaging in patients with metastasized ovarian and pancreatic cancer as part of the follow-up to therapy with [^90^Y]Y-FAPI-46. [^99m^Tc]Tc-FAPI-34 accumulated in the tumor lesions, as also demonstrated in PET/CT imaging using [^68^Ga]Ga-FAPI-46. However, the addition of amino acids with hydrophilic side chains led to only minor reductions in the logP value. Notably, in the case of arginine, the difference was most pronounced, and counterintuitively, the value increased in a substance with additional polar groups, which also exhibited better performance than the original FAPI-19. Therefore, other factors must account for the variations in tumor uptake. Chelating complexes of [^99m^Tc][Tc(CO)_3_]^+^ exhibit stability in terms of reaction kinetics but pose challenges in production, often leading to the formation of various complex isomers. This complexity hinders the efficiency of labeling the final product. Additionally, the need for precise pH adjustments during the radiolabeling process significantly limits its clinical suitability.

To overcome these issues, Ruan and co-worker in 2022 (Ruan et al. 2022) synthetized two ^99m^Tc-FAPI derivatives with the isonitrile group as a ligand to coordinate ^99m^Tc, with C_5_ and PEG_4_ chains as linkers to couple to the piperazine group of FAPI. In comparison to [^99m^Tc][Tc-(CN-C_5_-FAPI)_6_]^+^ (log P =  − 0.86 ± 0.03), [^99m^Tc][Tc-(CN-PEG_4_-FAPI)_6_]^+^ (log P =  − 2.38 ± 0.07) showed increased hydrophilicity, highlighting the significant role of the PEG_4_ linker in enhancing hydrophilicity. The in vitro cellular uptake results demonstrated a significantly high target affinity for FAP. Biodistribution and blocking studies revealed specific tumor uptake for both [^99m^Tc][Tc-(CN-PEG_4_-FAPI)_6_]^+^ and [^99m^Tc][Tc-(CN-C_5_-FAPI)_6_]^+^. Notably, [^99m^Tc][Tc-(CN-PEG_4_-FAPI)_6_]^+^ exhibited sixfold higher tumor uptake (8.05 ± 1.48% ID/g) then [^99m^Tc][Tc-(CN-C_5_-FAPI)_6_]^+^ (1.34 ± 0.13% ID/g) at 1h post-injection and a superior tumor/nontarget ratio, indicating its potential as a promising candidate for tumor imaging targeting FAP. Although this complex improved bioavailability and significantly increased tumor absorption, its limited water solubility remained a challenge, resulting in relatively high renal uptake. However, due to the relatively high uptake of [^99m^Tc][Tc-(CN-PEG_4_-FAPI)_6_]^+^ in nontarget tissues, such as the kidney and liver, SPECT/CT images are unsatisfactory.

With the aim to develop more water-soluble ^99m^Tc-FAPI complexes, Yang et al. (2023) synthesized and radiolabeled with technetium-99m HYNIC-Glc-FAPT, using tricine/EDDA or dimer tricine as co-ligands to produce [^99m^Tc]Tc-tricine/EDDA-HYNIC-Glc-FAPT ([^99m^Tc]Tc-TE-FAPT) and [^99m^Tc]Tc-tricine_(2)_-HYNIC-Glc-FAPT ([^99m^Tc]Tc-T_2_-FAPT) (Additional file 1: Fig. S4). Both radiopharmaceuticals were synthesized with high radiochemistry yield (> 97%) in 15 min, in a single-step procedure and they were stable in human serum. In comparison to [^99m^Tc]Tc-tricine_(2)_-HYNIC-Glc-FAPT, [^99m^Tc]Tc-tricine/EDDA-HYNIC-Glc-FAPT demonstrated a higher degree of hydrophilicity, characterized by a log P value of (− 3.53 ± 0.12). Tumor uptake of [^99m^Tc]Tc-TE-FAPT in U87 xenografted BALB/c nude mice at 1 h was 6.33 ± 2.10%ID/g, and the tumor/renal ratio was > 10 at all times above 1 h. Therefore, [^99m^Tc]Tc-TE-FAPT showed superior pharmacokinetic, tumor uptake, and retention capacity compared to previously reported ^99m^Tc-FAPI (Lindner et al. 2020b; Ruan et al. 2022; Trujillo-Benítez et al. 2022), but as the authors conclude, clinical trials are necessary to establish the relevance of these findings to the uptake of tumors in humans.

Very recently also Ruan and co-workers (Ruan et al. 2023a) utilized the ^99m^Tc-HYNIC labeling strategy to develop ^99m^Tc-FAPI derivates with improved specific tumor uptake, reduced uptake in non-target organs investigating also the impact of various linkers to finely adjust the physicochemical and biological characteristics of potential radiotracers. Specifically, on the base of hydrophilicity, low nonspecific binding affinity, and low toxicity, DPro-Gly linkers with varying lengths (n = 2 or 6 for the L1 or L2 ligand respectively) were employed to link with the HYNIC group and FAP molecule. [^99m^Tc]Tc-L1 and [^99m^Tc]Tc-L2 were then assessed through in vitro and in vivo studies to refine potential tumor imaging agents targeting FAP. Biodistribution studies in U87MG tumor-bearing mice showed that the absorption of [^99m^Tc]Tc-L1 in tumors is notably elevated (13.18 ± 1.26% ID/g), surpassing its uptake in other organs. Moreover, it exhibits favorable retention within the tumor within the initial hour. In contrast, the uptake of [^99m^Tc]Tc-L2 in tumors is comparatively lower (4.29 ± 0.22% ID/g), suggesting that the length of the linker DPro-Gly repeats has a substantial impact on the biodistribution performance. Additionally, [^99m^Tc]Tc-L2 demonstrates a relatively diminished target-to-nontarget ratio when compared to [^99m^Tc]Tc-L1. In comparison to the previously reported [^99m^Tc][Tc-(CN-PEG4-FAPI)_6_]^+^ (Ruan et al. 2022), the renal uptake of [^99m^Tc]Tc-L1 (5.22 ± 0.78%ID/g) is notably lower than that of [^99m^Tc][Tc-(CN-PEG4-FAPI)_6_]^+^ (12.98 ± 1.20%ID/g) at 1 h post-injection. Additionally, [^99m^Tc]Tc-L1 demonstrates a decrease in liver uptake from 4.90 ± 0.40%ID/g to 2.50 ± 0.77%ID/g. Importantly, the tumor uptake of [^99m^Tc]Tc-L1 (13.18 ± 1.26% ID/g) is substantially higher than that of [^99m^Tc][Tc-(CN-PEG4-FAPI)_6_]^+^ (8.05 ± 1.48%ID/g). This indicates that [^99m^Tc]Tc-L1 exhibits an enhanced specific tumor uptake and reduced non-target uptake. A kit formulation was created for easy labeling [^99m^Tc]Tc-L1, designed to be straightforward, effective, consistent, and suitable for clinical application. The resulting [^99m^Tc]Tc-L1 was tested in five healthy volunteers and three patients with various types of cancer, to confirm its safety and effectiveness (Ruan et al. 2023b), NCT05444686]. The kit-prepared [^99m^Tc]Tc-L1, utilized for imaging tumors associated with FAP, demonstrated robust in vitro stability and water solubility, along with notable tumor uptake, specific FAP binding, and favorable target-to-background (TBR) ratios. Assessment of its effective dose and biodistribution suggested its safety profile, with no discernible toxicities or adverse effects observed. While it exhibited high sensitivity in detecting primary tumors, additional research is needed to evaluate its efficacy in identifying metastatic lesions and for clinical staging purposes.

The labeling approach involving [^99m^Tc]Tc-HYNIC has been also employed by Trujillo-Benítez et al. in 2022. They presented a study involving the design, synthesis, and preclinical assessment of a fibroblast activation protein (FAP) inhibitor, utilizing boronic acid. Specifically, they utilized the ^99m^Tc-HYNIC-D-alanine-boroPro ([^99m^Tc]Tc-iFAP) as part of their investigative approach. The evaluation of biodistribution for [^99m^Tc]Tc-iFAP in nude mice bearing induced liver cancer tumors (Hep-G2 cells) revealed a tumor uptake of 7.05% and 5.18% of the % ID/g at 30 min and 2 h post-injection, respectively, with very rapid blood clearance. The elimination process primarily occurs through the kidneys.

SPECT Imaging with ^99m^Tc-Labeled HYNIC-FAPI-04 was recently reported by Luo and co-workers (Luo et al. 2023) to extend the differential time window in evaluating tumor fibrosis. The imaging efficacy of [^99m^Tc]Tc-HYNIC-FAPI-04 SPECT was assessed in mice bearing U87MG-derived FAP-positive tumors, further comparing it with [^68^Ga]Ga-FAPI-04 PET or [^18^F]F-FDG PET. At 1.5 h P.I., [^99m^Tc]Tc-HYNIC-FAPI-04 uptake was clearly observed in FAP-positive U87MG tumor at 2.67 ± 0.35% ID/mL. At 5 h P.I., U87MG tumor was still distinguishable with tracer uptake as high as 1.81 ± 0.20% ID/mL, demonstrating its potential as a suitable radiopharmaceutical for extending the differential time window.

The pro and cons of the most significant ^99m^Tc-FAPI labeled compounds discussed in this section are reported in Table 1. From the analysis of the results so far published in the literature it is evident that the HYNIC strategy currently appears to be the most utilized and promising in the development of a ^99m^Tc-labeled FAPI. In any case, to the best of our knowledge, an ideal ^99m^Tc-FAPI labeled compound is not yet available, and the optimization of labeled compounds for broader clinical utility in the future could potentially be achieved by evaluating the application of different synthetic strategies such as the “4 + 1” or “TcN” approaches (Fig. 2 and Fig. 3a).

**Table 1:** Characteristics of the most significant ^99m^Tc-FAPI labeled compounds

Author	Compound	Labelling strategy	Cell lines tested	Animal model	Pro	Cons
Roy et al. (2020)	[^99m^Tc]Tc-FL-L3	[^99m^Tc][TcO]^3+^	Embryonic kidney HEK 293	MDA-MB231 breast tumors	Minimal uptake in non-target tissues	Relatively low tumor uptake
Lindner et al. (2020b)	[^99m^Tc]Tc-FAPI-34	[^99m^Tc][Tc(CO)_3_]^+^	HT-1080-FAP; HEK-muFAP; HEKCD26	HT-1080-FAP–xenotransplanted mice	Stability in terms of reaction kinetics; low liver uptake	Formation of various complex isomers; need for precise pH adjustments during the radiolabeling process significantly limits clinical suitability
Ruan et al. (2022)	[^99m^Tc][Tc-(CN-PEG_4_-FAPI)_6_]^+^	[^99m^Tc]Tc-isonitrile	HT-1080-FAP	U87 xenografted BALB/c nude mice	High tumor uptake	Limited water solubility; relatively high uptake in nontarget tissues, such as the kidney and liver
Trujillo-Benítez et al. (2022)	[^99m^Tc]Tc-iFAP	[^99m^Tc]Tc-HYNIC	N30 (FAP +)	Mice bearing induced liver cancer tumors (Hep-G2 cells)	Very rapid blood clearance; high sensitivity	Formation complex isomers; labeling with ^188^Re is not feasible
Yang et al. (2023)	[^99m^Tc]Tc-TE-FAPT	[^99m^Tc]Tc-HYNIC	A549-FAP	U87 xenografted BALB/c nude mice	Tumor/renal ratio > 10	Formation complex isomers; labeling with ^188^Re is not feasible
Ruan et al. (2023a)	[^99m^Tc]Tc-L1	[^99m^Tc]Tc-HYNIC	HT-1080-FAP	Mice bearing U87MG-derived FAP-positive	Favorable target-to-background ratios; robust in vitro stability and water solubility	Formation complex isomers; labeling with ^188^Re is not feasible
Luo et al. (2023)	[^99m^Tc]Tc-HYNIC-FAPI-04	[^99m^Tc]Tc-HYNIC	U87MG cells (FAPI +)	Mice bearing U87MG-derived FAP-positive	Sensitive to tumor progression	Formation complex isomers; labeling with ^188^Re is not feasible

## Clinical applications in oncology

The results of the first clinical studies with ^99m^Tc-labeled FAPI tracers have been summarized in Table 2.

**Table 2:** Clinical applications of [^99m^Tc]Tc-FAPI tracers in oncology

Author	Year/country	Compound	Administered activity	Imaging Protocol	Clinical setting	Patient cohort	Comment
Jia et al. (2023)	2023/China	FAPI-04	790.4–930.2 MBq	Biodistribution: WB at 10, 30, 90, 150 and 240 min (n = 4) Diagnostics: WB + SPECT/CT at 60–90 p.i. (n = 40)	Digestive system tumors	40	[^99m^Tc]Tc-HFAPi SPECT/CT showed high diagnostic performance, superior to that of ce-CT in the assessment of distant metastases (in particular liver and bone)
Ruan et al. (2022, 2023a, 2023b)	2023/China	DP-FAPI	828.8–1128.5 MBq	Biodistribution: WB at 0, 1, 2, 4, 8, and 24 h Diagnostics: SPECT/CT at 2 h	Gastrointestinal tumors	7 (4 healthy volunteers and 3 oncological)	[^99m^Tc]Tc-DP-FAPI was well-tolerated by patients and exhibited high diagnostic performance for the detection of gastrointestinal tumors
Coria-Domínguez et al. (2022)	2022/Mexico	iFAP	740 MBq	Biodistribution: WB at 0.5, 2, 4 and 24 h Diagnostics: SPECT/CT at 3 h	healthy volunteers; cervical, lung and breast cancer	9 (6 healthy volunteers and 3 oncological)	[^99m^Tc]Tc-iFAP uptake intensity (cervical > breast > lung) resulted inverse to that of [^18^F]FDG (lung > breast > cervical)
Vallejo-Armenta et al. (2022)	2022/Mexico	iFAP	735 ± 63.5 MBq	Diagnostics: SPECT/CT at 2 h	5 brain tumors (gliomas) and 27 tumors of various districts	32	[^99m^Tc]Tc-iFAP SPECT/CT detected less metastases than [^18^F]FDG PET/CT, but showed good performances in the discrimination of high-grade vs low-grade gliomas

**Fig. 5:**
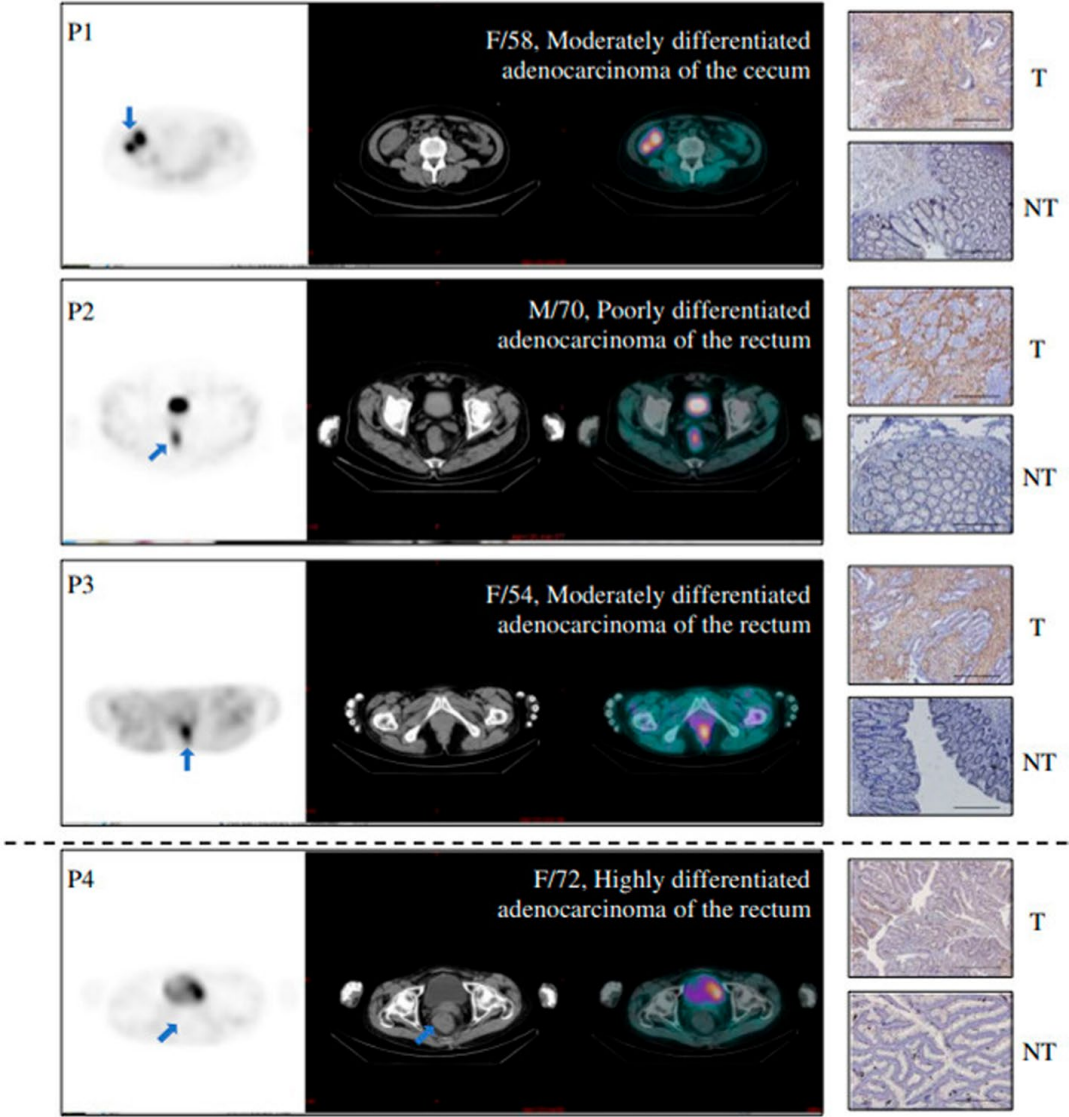
The figure shows the direct comparison of the uptake at [^99m^Tc]Tc-HFAPi SPECT/CT (left) and immunohistochemistry staining (right) in primary digestive-tract tumors. The last case (P4) refers to a highly differentiated rectal adenocarcinoma tumor that resulted false negative at [^99m^Tc]Tc-HFAPi SPECT/CT. Scale bar, 200 μm; T: pri- mary tumour; NT: Tumour-adjacent tissue. Reprinted from (Jia et al., 2023), under a Creative Commons Attribution 4.0 International License (http://creativecommons.org/licenses/by/4.0/). No changes were made

In a recently published paper, Jia et al. (Jia et al. 2023) investigated the biodistribution and diagnostic value of [^99m^Tc]Tc-FAPI-04 ([^99m^Tc]Tc-HFAPi) in 40 patients with suspected or confirmed tumors of the digestive tract. Biodistribution studies were conducted in four cases through whole-body planar imaging at 10, 30, 90, 150, and 240 min post-injection. For all other patients, whole-body scans and SPECT/CT were obtained 60–90 min after tracer administration. Among the enrolled patients, thirty were therapy-naïve, five had been previously treated with chemo/radiotherapy, while the remaining five cases had undergone surgery with or without chemo/radiotherapy. At the final analysis, thirty-nine cases had confirmed malignant lesions, while one patient was affected by a benign lesion (i.e., spindle cell tumor of the intestine). In all cases, [^99m^Tc]Tc-HFAPi administration was well-tolerated, and the biodistribution study showed physiological tracer accumulation in the liver, pancreas, gallbladder, and to a lesser extent in the kidneys, lungs, spleen, salivary glands, and thyroid glands, with rapid clearance of the radiotracer from these organs. The mean effective dose equivalent of the whole body was 1.26 × 10^−3^ mSv/MBq. As for the diagnostic performance in primary tumors, [^99m^Tc]Tc-HFAPi SPECT/CT exhibited high sensitivity (94.2%), identical to that of contrast-enhanced computed tomography (ceCT), with 2 false-negative cases in highly differentiated rectal adenocarcinoma. Notably, [^99m^Tc]Tc-HFAPi uptake correlated with FAP expression, as the false-negative cases exhibited the lowest level of FAP (Fig. 5). Of note, gastric and colon cancers were associated to the most intense uptake at [^99m^Tc]Tc-HFAPi SPECT/CT (median TBR and SUVmax of 7.01 and 6.35, and 12.43 and 9.13, respectively), consistently with [^68^Ga]Ga-FAPI-04 PET/CT reports (Pang et al. 2021).

Regarding metastatic lesions, [^99m^Tc]Tc-HFAPi detected 26 lesions in 8 patients, including liver (n = 15), bone (n = 8), abdomen (n = 1), pelvic (n = 1), and mediastinal tissue (n = 1) with higher sensitivity and specificity than ceCT, except for a single false-positive case due to tuberculosis. It is worth mentioning that [^99m^Tc]Tc-HFAPi demonstrated particularly high diagnostic accuracy (sensitivity 88.2%, specificity 100%) for detecting liver metastases and revealed more bone metastases than ceCT in 2 patients with diffuse skeletal spread. The authors also assessed the impact of [^99m^Tc]Tc-HFAPi SPECT/CT on patients’ clinical management. According to [^99m^Tc]Tc-HFAPi SPECT/CT, TNM stage and oncologic management was changed in 20% and 15% of patients, respectively. Tracer uptake was also evaluated using a quantitative approach, both by calculating the maximum standardized uptake value (SUVmax) and the tumor-to-background ratio (TBR). ROC analysis was conducted to identify optimal cut-off values to discriminate between malignant and benign lesions, yielding an AUC of 0.938 for the T/B ratio and 0.913 for SUVmax.

A very recently preliminary clinical study (Ma et al. 2023, NCT05859763) conducted in breast cancer patients revealed that [^99m^Tc]Tc-HYNIC-FAPI-04 demonstrated a notable advantage over ^18^F-FDG (fluorodeoxyglucose) in detecting lymph node metastatic lesions. This finding is significant as it can potentially enhance treatment strategies for breast cancer patients. Specifically, [^99m^Tc]Tc-HYNIC-FAPI-04 exhibited excellent affinity and specificity for FAP, indicating its promise as a single photon emission computed tomography (SPECT) radiotracer. Its effectiveness suggests potential applications in the (re)staging and treatment planning of breast cancers.

Another FAPI-derived ligand (DP-FAPI), incorporating d-proline as a linker, was meticulously designed and synthesized by Ruan and colleagues (Ruan et al. 2023b). The resulting hydrophilic ^99m^Tc-labeled complex ([^99m^Tc]Tc-DP-FAPI) exhibited remarkable stability. Post-radiolabeling via kit formulation, the radiochemical purity of the product exceeded 95%, allowing for subsequent experiments without the need for purification. This synthesized compound underwent in vitro testing and evaluation in mice bearing FAP-positive U87 MG tumors through micro-SPECT. In animal models, tumor uptake (16.26 ± 2.71%ID/g) demonstrated a notable increase compared to other organs, with robust retention in the tumor observed within 4 h (8.02 ± 0.86%ID/g). Importantly, an inhibition experiment using cold FAP-ligand revealed a significant 88% reduction in tracer uptake, indicating the specificity of [^99m^Tc]Tc-DP-FAPI binding to the target. For biodistribution analysis, healthy volunteers received injections with an activity ranging from 828.8 to 1128.5 MBq of [^99m^Tc]Tc-DP-FAPI, followed by serial whole-body scans at 0, 1, 2, 4, 8, and 24 h. Additionally, quantitative SPECT/CT at 2 h post-injection was conducted in three patients with gastrointestinal tumors (pancreatic adenocarcinoma, signet-ring cell gastric carcinoma, intrahepatic cholangiocarcinoma). The radiopharmaceutical demonstrated favorable tolerance in both the healthy control groups and oncological patients. Furthermore, FAPI SPECT/CT exhibited excellent diagnostic performance, characterized by high contrast images, as evidenced by SUVmax values ranging from 3.22 to 11.33 and a TBR (using non-pathological liver as a reference) spanning 1.58 to 3.58.

In a feasibility study, after testing [^99m^Tc]Tc-iFAP SPECT/CT on healthy volunteers, Coria-Domínguez and colleagues performed and compared [^99m^Tc]Tc-iFAP SPECT/CT with [^18^F]FDG PET/CT in three oncologic patients affected by cervical (squamous cell), breast (triple-negative) and lung (adenocarcinoma) cancer, respectively (Coria-Domínguez et al. 2022). [^99m^Tc]Tc-iFAP SPECT/CT images acquired 1–3 h after radiotracer injection showed optimal diagnostic quality, with good TBR. Of note, [^99m^Tc]Tc-iFAP and [^18^F]FDG showed inverse uptake intensity among the three different oncological malignancies. In particular, cervical cancer showed higher uptake at [^99m^Tc]Tc-iFAP, lung cancer resulted more [^18^F]FDG-avid and breast cancer showed intermediate affinity for the 2 radiotracers. Primary tumors showed significantly higher uptake than lymph node metastases, in both breast and lung cancer patients.

In another study (Vallejo-Armenta et al. 2022), the same group of authors imaged 32 patients with different primary cancers 2 h post injection of 735 ± 63.5 MBq of [^99m^Tc]Tc-iFAP. No adverse effects were reported after radiotracer injection. Apart from the patients affected by gliomas, who received cranial MRI as standard of reference imaging, [^18^F]FDG PET/CT was performed in all patients investigated and the results of the 2 imaging were compared. Of note, [^99m^Tc]Tc-iFAP SPECT/CT detected 100% of primary tumors. Among breast cancer patients, the uptake was particularly intense in HER2-enriched tumors. Nevertheless, [^99m^Tc]Tc-iFAP SPECT/CT was outperformed by [^18^F]FDG PET/CT when considering lymph node and distant metastases detection (51% and 17% vs 100% and 88%, respectively), except for a patient with peritoneal carcinomatosis secondary to colorectal cancer, that was detectable only at [^99m^Tc]Tc-iFAP SPECT/CT. Among undetected lymph node and distant metastases at [^99m^Tc]Tc-iFAP, 39% had a size > 8mm and were, therefore, potentially detectable at SPECT imaging.

Interestingly, [^99m^Tc]Tc-iFAP showed good performances in the discrimination of high-grade (4/4 SPECT positive) vs low-grade gliomas (1/1 SPECT negative), due to the optimal contrast of lesions versus low background activity. Nevertheless, these preliminary findings need to be confirmed in studies with larger sample size.

In conclusion, despite several ^99m^Tc-labeled FAPI radicompounds have been used in published papers, they generally showed high diagnostic performances in oncological patients, superior to those of ceCT and at least similar to those of [^18^F]FDG PET/CT. ^99m^Tc-labeled FAPI radicompounds may contribute mainly in patients affected by gastrointestinal and breast cancers, with further investigation needed also in other oncological diseases (i.e. the discrimination of high-grade vs low-grade gliomas).

## Non-oncological applications

Table 3 schematizes the first clinical applications of ^99m^Tc-labeled FAPI imaging in non-oncological patients.

**Table 3:** Applications of [^99m^Tc]Tc-FAPI tracers in inflammation

Author	Year/country	Compound	Administered activity	Imaging protocol	Clinical setting	Patient cohort	Comment
Liu et al. (2023a, b, )	2023/China	HFAPI	695.6–888.0 MBq	Biodistribution: WB at whole-body scintigraphy at 5 min, 20 min, 40 min, 1 h, 2 h, 3 h, 4 h, and 6 h Diagnostics: chet SPECT/CT at 4 h	Idiopathic pulmonary fibrosis	11	[^99m^Tc]Tc-HFAPI SPECT/CT showed promising results for detecting and scoring IPF severity, with good correlation with HRCT and clinical parameters
Xie et al. (2023)	2023/China	HFAPI	777 ± 140 MBq	Cardiac SPECT/CT at 195 ± 20 min	Early myocardial fibrosis	106 (20 gender-matched healthy controls)	[^99m^Tc]Tc-HFAPI SPECT might have role of detect early-stage myocardial fibrosis in hypertensive patients

**Fig. 6:**
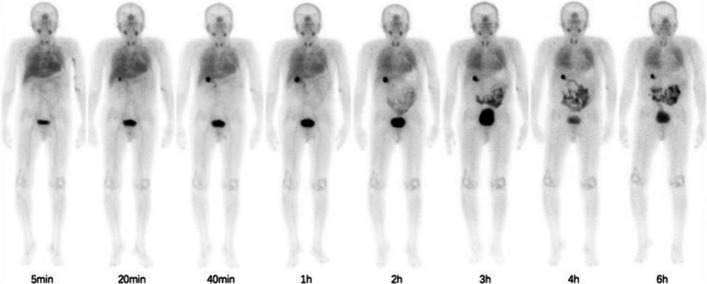
Serial whole-body scans acquired in a representative patient affected by idiopathic pulmonary fibrosis at different time-points. Note the physiological distribution of the tracer in the liver, intestinal tract, pancreas, gallbladder and spleen and the pathological uptake in both the lungs. Reprinted from (Liu et al. 2023b), under a Creative Commons Attribution 4.0 International License (http://creativecommons.org/licenses/by/4.0/). No changes were made

**Fig. 7:**
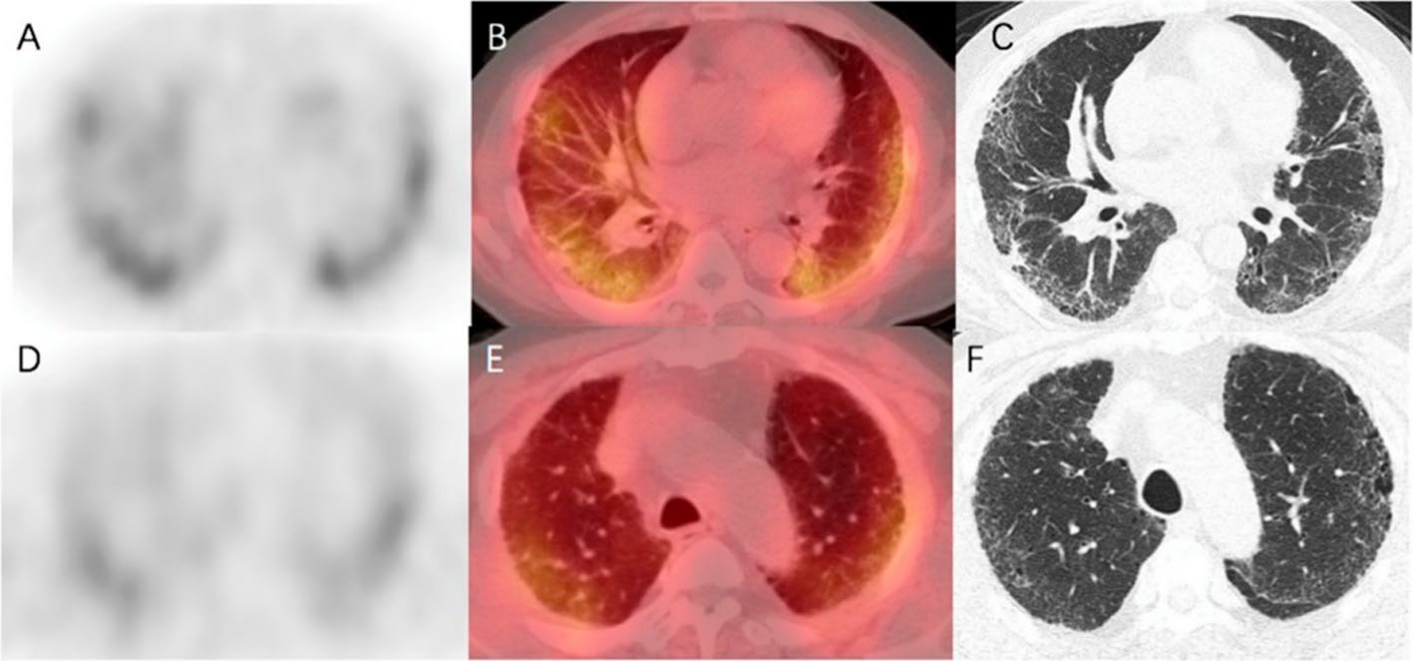
Fused axial SPECT/CT images in a patient affected by idiopathic pulmonary fibrosis. Upper row: corresponding emissive **A**, fused **B** and transmissive **C** slices of the lower left and right pulmonary lobes; note the intense tracer incorporation. Lower row: lower tracer incorporation can be observed in the corresponding emissive **D**, fused **E** and transmissive **F** slices of the middle-upper region of the lungs. Reprinted from (Liu et al. 2023b), under a Creative Commons Attribution 4.0 International License (http://creativecommons.org/licenses/by/4.0/). No changes were made

Fibroblast activation is identified as a key histological feature in interstitial lung diseases (ILD), correlating with disease severity and mortality, particularly in the case of idiopathic pulmonary fibrosis (IPF), the most prevalent form of ILD. FAP-targeted imaging offers a unique opportunity to gain insight into the physiopathological processes of IPF. In this context, Xie et al. assessed the distribution and potential clinical utility of [^99m^Tc]Tc-HYNIC-FAPI-04 ([^99m^Tc]Tc-HFAPI) in 11 therapy-naïve IPF patients who had previously undergone high-resolution CT (HRCT) for the assessment of the usual interstitial pneumonia (UIP) pattern (Liu et al. 2023a). Following the injection of an activity range of 695.6–888.0 MBq, seven representative patients underwent a biodistribution study through serial whole-body scans at 5 min, 20 min, 40 min, 1 h, 2 h, 3 h, 4 h, and 6 h. Additionally, chest SPECT/CT at 4 h was performed for the qualitative and quantitative analysis of IPF. In this regard, the grade of tracer uptake in the affected lung was quantified using SUVmax and lesion-to-background ratio, with the blood pool as a reference. Normal physiological uptake of [^99m^Tc]Tc-HFAPI was primarily observed in the liver, intestinal tract, pancreas, gallbladder, and, to a lesser extent, in the spleen, kidneys, and thyroid, with rapid clearance and a whole-body effective dose of 0.0041 ± 0.000559 mSv/MBq per patient (Fig. 6). Increased and prolonged tracer incorporation was noted in the affected lungs, still detectable at 6 h post-injection, and correlated with the severity of IPF and pulmonary function tests. The higher the severity of interstitial disease, as scored on HRCT using a 5-point scale, the higher the SUV and LBR values, except for score 4 (Fig. 7). The lack of correlation between tracer uptake grade (SUV, LBR) and disease severity in HRCT score 4 IPF was explained by the authors, who suggested that massive vesicles in the honeycombing and reticular opacity regions of HRCT stage 4 might reduce tracer signal intensity. Importantly, the findings from [^99m^Tc]Tc-HFAPI SPECT/CT were substantially in line, both qualitatively and quantitatively, with those obtained using [^68^Ga]Ga-FAPI-04 (Bergmann et al. 2021).

Hypertensive myocardial fibrosis presents a complex clinical challenge, with pathogenetic mechanisms intricately tied to fibroblast proliferation (Berk et al. 2007). This proliferation occurs in response to heightened ventricular wall stress, leading to the synthesis and deposition of proteins in the extracellular matrix. While endomyocardial biopsy remains the gold standard for diagnosis, its widespread application is hindered by its invasive nature. As an alternative to biopsy, certain biomarkers associated with myocardial fibrosis, such as procollagen Type I N-Terminal propeptide and C-Terminal Telopeptide of Collagen Type I, have shown promise (Ding et al. 2020). However, their primary limitation lies in a lack of specificity. In terms of imaging approaches, both echocardiography and cardiac magnetic resonance (CMR) have been employed with encouraging results, although they may fall short in detecting early-stage fibrosis (Gupta et al. 2021). A recent investigation conducted by Xie and colleagues sought to explore the potential of [^99m^Tc]Tc-HFAPI for early myocardial fibrosis detection in hypertensive hearts. The study was divided into a preclinical and clinical phase. In the preclinical experiments, spontaneously hypertensive rats (SHRs) and age-matched Wistar Kyoto rats (WKYs) were randomly assigned to three groups (8, 16, and 28 weeks) and subjected to [^99m^Tc]Tc-HFAPI and echocardiography. According to the results of autoradiography and histological analysis conducted on the left ventricle, mRNA and protein levels of FAP in SHRs began to increase from week 8, while changes in collagen I levels were not detected until week 28. In the clinical study, 106 patients with essential hypertension and 20 gender-matched healthy controls were recruited. In all cases, an activity of 777 ± 140 MBq of [^99m^Tc]Tc-HFAPI was administered, and cardiac SPECT was performed at 195 ± 20 min post-injection. Images were scored from 0 to 2 based on the degree of tracer uptake (0 = no uptake, 1 = mild uptake above background; 2 = intense uptake). Additionally, SPECT images were processed using the QPS software, and polar maps were generated. Increased [^99m^Tc]Tc-HFAPI uptake was observed in 59 patients (scored as mild and intense in 56% and 22% of cases, respectively) and in 2 healthy controls. It is noteworthy that abnormal [^99m^Tc]Tc-HFAPI incorporation was also detected in patients with normal diastolic indicators (34%), normal left ventricular geometry (41%), and normal global longitudinal strain (20%), suggesting that early fibrogenesis precedes morphological changes.

To summarize, ^99m^Tc-labeled FAPI may play a role also in non-oncological diseases, mainly those correlated to fibrosis. [^99m^Tc]Tc-HFAPI has been investigated and showed promising results in IPF – with the potential to diagnose and stratify the severity of the disease – and myocardial fibrosis – contributing to the early detection of disease.

## Future perspectives of [^99m^Tc]Tc-FAPI imaging for clinical use: challenges and potential

As for the applications of ^99m^Tc-labeled radiocompounds in cancer, relevant considerations can be drawn from the analysis of existing literature. The administration of [^99m^Tc]Tc-FAPI compounds was generally well-tolerated by patients, with no significant adverse effects, and imaging was feasible with an excellent tumor-to-background ratio. When compared to contrast-enhanced CT (ceCT), [^99m^Tc]Tc-FAPI SPECT/CT showed similar diagnostic accuracy in gastrointestinal tumors, with higher sensitivity in the case of liver metastases (Jia et al. 2023). Notably, [^99m^Tc]Tc-FAPI imaging holds particular promise in the case of gastric signet ring cell adenocarcinoma, known for its low FDG avidity (Dondi et al. 2021). From a technical perspective, while a homogeneity of the administered activity (around 740 MBq) was observed across various clinical research papers, some heterogeneity was evident regarding the optimal time point for imaging, ranging from 1 to 3 h post-injection. An alarm bell has been ringing during the study by Vallejo-Armenta et al. (Vallejo-Armenta et al. 2022). The authors state that [^18^F]FDG PET/CT detected a significant higher number of lymph node and distant metastases than [^99m^Tc]Tc-FAPI SPECT and a considerable quote (39%) of those had a size above the resolution power of SPECT imaging. Indeed, this data is very preliminary and collected in a heterogenous spectrum of oncological disease. These findings appear to contradict previously reported data indicating a higher sensitivity of FAP-targeted imaging for detecting distant metastases compared to [^18^F]FDG (Liu et al. 2023a, b ). However, it should be noted that FAP-positivity in metastases depends on the grade of tumor invasion, stroma reaction, and specific histotypes. In this regard, a higher sensitivity of [^18^F]FDG PET/CT for lymph node detection compared to FAP-based imaging has been described in head and neck cancer cases (Gu et al. 2024). Regarding breast cancer specifically, scientific applications of FAPI PET/CT are still very limited and heterogeneous in terms of clinical setting and tumor phenotype (luminal A or B, HER2 positive, or triple negative), often conducted on small cohorts of patients, although FAPI PET generally detected more numerous lesions than [^18^F]FDG (Evangelista et al. 2023). Therefore, further investigations are required to more clearly define whether the data reported by Vallejo-Armenta and collaborators might be attributed to the well-known limited spatial resolution of SPECT compared to PET technology or to still unexplored biological issues.

The theranostic implications of FAPI-targeted imaging represent a compelling facet of this approach. In essence, theranostics entails a cutting-edge integrated platform involving the sequential administration of two nearly identical compounds. The first compound is labeled with a radionuclide suitable for imaging with SPECT or PET, while the second is conjugated with a radionuclide emitting beta or alpha particles, exerting anti-tumor effects (Morgan et al. 2023). It is postulated that FAPI-positive tumors could potentially benefit from theranostic approaches, utilizing ^90^Y/^177^Lu-labeled FAPI-targeting compounds, along the trajectory of the successful experiences obtained in neuroendocrine, hepatic tumors, and prostate cancer (Filippi et al. 2020; Uccelli et al. 2021). A recent report involving 21 patients (sarcoma, n = 16; pancreatic cancer, n = 3; prostate, n = 1; gastric cancer, n = 1) indicated the safety of [^90^Y]Y-FAPI-46 radioligand therapy, with one case showing a partial response and approximately one-third of patients experiencing stable disease (Fendler et al. 2022). However, the cited paper utilized FAPI-PET for assessing patients' eligibility through SUVmax measurements. Notably, ^99m^Tc-labeled FAPI tracers have yet to be validated for theranostic applications, as far as our knowledge extends. With a theranostic purpose, [^99m^Tc]Tc-FAPI-34 has an advantage over [^99m^Tc]Tc-iFAP, because the former is based on the tricarbonyl labeling technology, which is proven for use with ^188^Re, while with HYNIC, the labeling with ^188^Re is not feasible.

Beyond oncology, FAPI-based imaging is garnering attention from the scientific community for assessing infectious and inflammatory processes (Treglia and Albano 2023). Historically, the 'gold standard' nuclear medicine technique for infection evaluation has been the white blood cell (WBC) scan, preferably conducted using SPECT/CT technology (Filippi and Schillaci 2006). Both [^18^F]FDG and [^67^Ga]Ga-citrate have proven effective for imaging chronic inflammatory conditions like sarcoidosis or granulomatosis, although impaired, respectively, by specificity and dosimetric limitations. Preliminary clinical results with ^99m^Tc-labeled tracers have shown promising outcomes for the imaging of several inflammatory conditions. Specifically, the degree of tracer uptake, as determined by SUVmax, has been correlated with disease severity and, albeit moderately, with clinical symptoms in the case of interstitial lung disease, aligning with previously published reports utilizing [^68^Ga]Ga-FAPI PET (Liu et al. 2023b). Particularly promising are the applications of FAPI-based imaging in hypertensive myocardial fibrosis. In light of the socio-demographic impact of cardiovascular disease, the potential of FAPI tracers for patient stratification through early detection of myocardial fibrotic changes is capturing the attention of the scientific community. While the majority of published papers focus on the role of FAPI-PET tracers, scientific evidence regarding ^99m^Tc-based FAPI imaging remains limited (Barton et al. 2023). The paper by Xie and colleagues represents a highly innovative approach, conducted on a relatively large cohort of patients, supporting the use of [^99m^Tc]Tc-FAPI imaging in this specific clinical setting (Xie et al. 2023). It is noteworthy that SPECT is traditionally employed for myocardial perfusion imaging, often utilizing dedicated software for image processing, due to its more accessible cost and widespread diffusion. In this context, [^99m^Tc]Tc-FAPI SPECT might emerge as the modality of choice in this unique clinical setting. Further investigations should focus on exploring this topic.

Despite the great potential of [^99m^Tc]Tc-FAPI SPECT for several oncologic and non-oncologic applications, a few additional limitations should be considered besides the paucity of literature data available. In particular, the wide heterogeneity of the radiolabeled FAPI tracers that has been investigated hinders a comprehensive point of view, as different FAPI compounds may have different affinity for FAP expressed in different clinical conditions.

Moreover, a direct comparison of [^99m^Tc]Tc-FAPI SPECT/CT and [^68^Ga]Ga-FAPI PET/CT is still missing. Indeed, PET imaging guarantees higher spatial resolution and is supposed to be more accurate than SPECT. However, a direct comparison between the two imaging modalities is desirable to define the true extent of SPECT imaging "underpowering".

## Conclusions

Several approaches have been investigated for labeling FAPi-based compounds with ^99m^Tc, as SPECT imaging is still cheaper and more diffuse worldwide than PET imaging. Preliminary data showed promising results in terms of specificity for FAP-positive cells and, as a consequence, for diagnostic performances for several oncological and non-oncological conditions. An effort to find more hydrophilic FAPi-based compounds is desirable, as they could improve pharmacokinetic properties and achieve a more favorable biodistribution, enhancing the detection rate in particular in the abdomen region.

### Supplementary Information

**Additional file 1**. Displays additional figures on different coordination geometry of ^99m^Tc-radiopharmaceuticals; reaction scheme for the labeling of a biomolecule with ^99m^Tc; chemical structure of [^99m^Tc]Tc-FAPI-L3, [^99m^Tc]Tc-TE-FAPT, [^99m^Tc]Tc-T(2)-FAPT and research strategy.

## Data Availability

'Not applicable'.

## Abbreviations

CMR: Cardiac magnetic resonance
CNR: Isocyanide derivatives
Cys: Cysteine
DADS: Diamido-dithiols
DADT: Diamino-dithiols
DP: D-proline
EDDA: Ethylenediamine-N,N'-diacetic acid
EDTA: Ethylenediaminetetra-acetic acid
FAP: Fibroblast activating protein
FAPi: FAP inhibitors
FDG: Fluorodeoxyglucose
HEK: Human embryonic kidney
his: Histidine
HRCT: High-resolution CT
HYNIC: 6-Hydrazinopyridine-3-carboxylic acid
ILD: Interstitial lung diseases
IPF: Idiopathic pulmonary fibrosis
LBR: Lesion-to-background ratio
MAG3: Mercaptoacetyltriglycine
MAMA: Monoamino-monoamido-dithiols
PCa: Prostate cancer
PEG: Polyethylene glycol
PET: Positron emission tomography
PNP: Diphosphinoamine
PR_3_: Tertiary phosphine
PSMA: Prostate specific membrane antigen
RCP: Radiochemical purity
SHRs: Spontaneously hypertensive rats
SPECT: Single photon emission computed tomography
SUV: Standardized uptake value
TBR: Target-to-background ratio
UIP: Usual interstitial pneumonia
WKYs: Wistar Kyoto rats
