# Supplementary material for: 99mTc-labeled FAPI compounds for cancer and inflammation: from radiochemistry to the first clinical applications
Source: EJNMMI Radiopharm Chem. 2024 May 2;9:36. doi: 10.1186/s41181-024-00264-0 (PMC11065808; doi:10.1186/s41181-024-00264-0)
Supplement: Supplementary file 1 — Additional file 1. Displays additional figures on different coordination geometry of 99mTc-radiopharmaceuticals; reaction scheme for the labeling of a biomolecule with 99mTc; chemical structure of [99mTc]Tc-FAPI-L3, [99mTc]Tc-TE-FAPT, [99mTc]Tc-T(2)-FAPT and research strategy. [file 41181_2024_264_MOESM1_ESM.pdf]

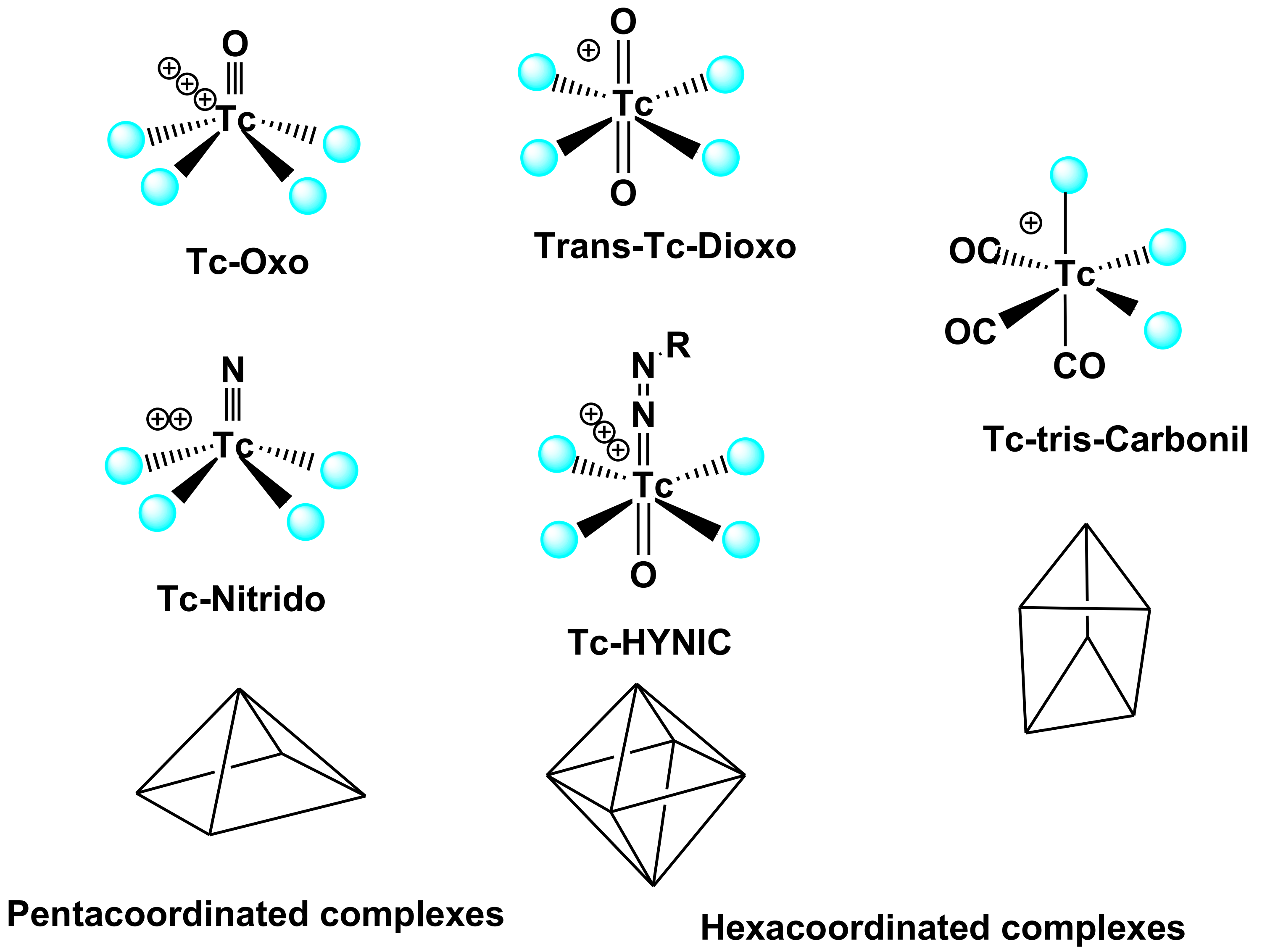

Figure S1. Different coordination geometry of ^{99m}Tc-radiopharmaceuticals

$$[^{99m}\mathrm{Tc}]\mathrm{TcO_4}^- + \mathrm{Sn}^{2+} + \text{L-Biomolecule} \longrightarrow [^{99m}\mathrm{Tc}]\mathrm{Tc}\text{-(L-Biomolecule)}_n + [\,\mathrm{Sn(OH)}_m]^{4-m}$$

Figure S2. Reaction scheme for the labeling of a biomolecule with ^{99m}Tc. L-Biomolecule represents a suitable ligand, called “bifunctional ligand” because it can coordinate with the metal and simultaneously bind to the biomolecule of interest. The number “n” and therefore the number of biomolecules present in the final complex depends on the type of ligand L and the synthetic strategy used

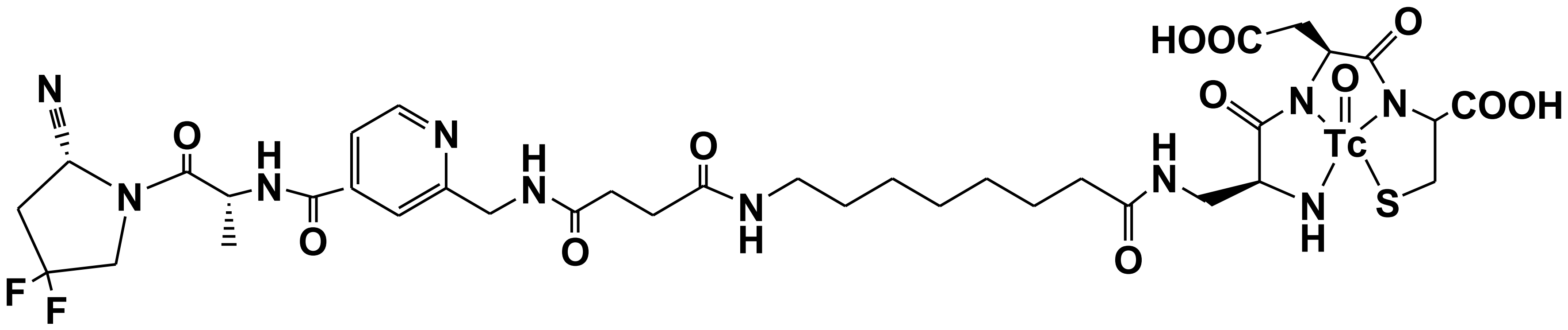

Figure S3. Chemical structure of [^{99m}Tc]Tc-FAPI-L3

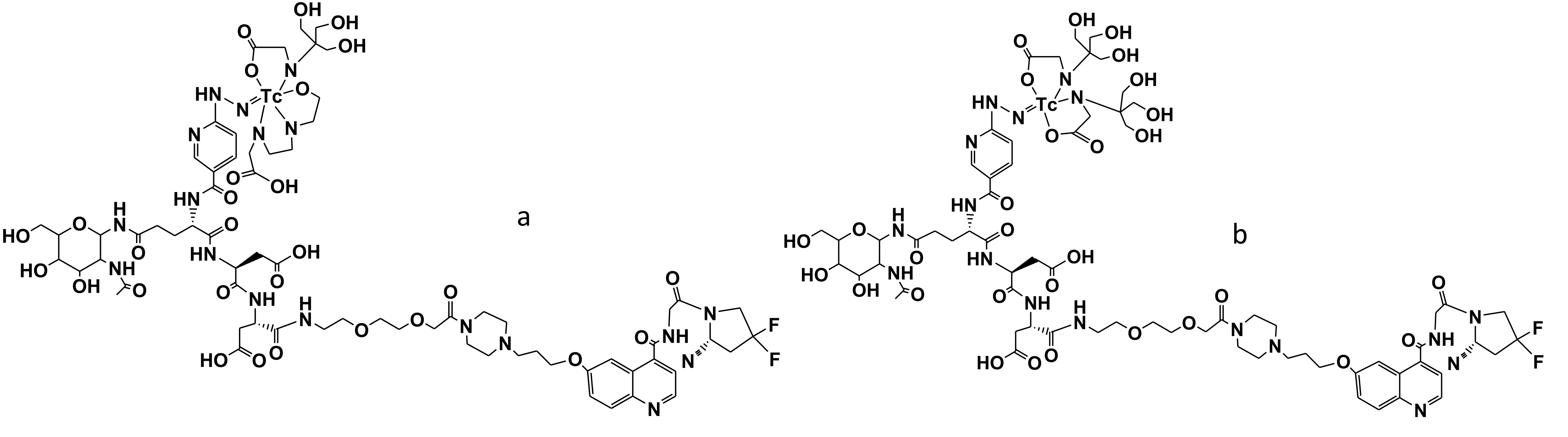

Figure S4. (a) Chemical structure of $[^{99m}\text{Tc}]$Tc-TE-FAPT, (b) $[^{99m}\text{Tc}]$Tc-T(2)-FAPT

Search strategy and study selection: A literature search up to January was conducted by searching the PubMed, Scopus, Google Scholar, and Web of Science databases. The search terms used were as follows: “technetium 99m” or “99mTc” AND “fibroblast activation protein” or “FAPI”. The search was performed both with and without the addition of filters, such as limiting to English language only and specific article types (original article, research article). Original research papers in both preclinical and clinical fields were considered eligible. For clinical research papers, no restrictions were placed on the sample size. Reviews (including systematic reviews with or without meta-analysis), case reports, meeting abstracts, and editorials were excluded. Two reviewers (LUc and LF) conducted the literature search, while two other reviewers (PM and LUr) independently selected the studies to consider, excluding duplicate papers. Any discrepancies were resolved through consensus. After compiling all identified records, full texts were retrieved and further assessed by a fourth reviewer (AB), who also checked the references of the selected studies to ensure their eligibility.

Data extraction: General details were retrieved for each study, including generic data (authors, journal name, year of publication, country, and study design). For preclinical studies, technical data regarding the labeling procedure, the type of radiocompound obtained, its pharmacokinetics and dynamics, and the testing modality were retrieved and reported in the manuscript. For clinical studies, information such as the clinical setting (oncological or non-oncological), the number of patients, the employed radiopharmaceutical, any comparison with a different tracer or imaging modality, and the sample size of included patients were retrieved and reported in the manuscript.
